# Prognostic significance vestibular examination results in patients with vestibular migraine

**DOI:** 10.3389/fneur.2024.1370940

**Published:** 2024-04-10

**Authors:** Fumiyuki Goto, Koichiro Wasano, Shoji Kaneda, Kenji Okami

**Affiliations:** Department of Otolaryngology, School of Medicine, Tokai University, Isehara, Kanagawa, Japan

**Keywords:** migraine, dizziness, prophylaxis, clinical improvement, prediction

## Abstract

**Introduction:**

Vestibular migraine (VM) is a newly defined clinical condition. Several vestibular abnormalities have been reported in patients with VM. However, to date, no specific vestibular examinations are used to define VM. Therefore, the utility of vestibular examinations is limited. Currently, the role of vestibular examination has not been clearly defined. We speculated that the results of vestibular examinations could predict the prognosis of VM. We investigated the relationship between the vestibular examination results and clinical outcomes in patients with VM.

**Methods:**

This study included 25 patients with VM. Vestibular examinations, including the video head impulse test (V-HIT), cervical and ocular vestibular evoked myogenic potential (c-VEMP and o-VEMP), posturography, and several questionnaires, including the Dizziness Handicap Inventory (DHI), were conducted at the initial evaluation. Lifestyle modifications for VM and conventional pharmacological prophylactic treatments, including lomerizine, amitriptyline, and valproic acid, were performed. After 4 weeks of treatment, clinical improvements were evaluated using the Clinical Global Improvement Scale (CGI-s). The relationships among the CGI-S score, several clinical variables, and the results of several vestibular examinations were evaluated. Each patient was further classified into two subgroups according to treatment outcomes concerning vertigo and headache: CGI-S score from 0 to 2 (good response [GR]) and CGI-S score > 3 (poor response [PR]).

**Results:**

Overall, after treatment, most of the patients had improved dizziness and headache, and the CGI-s was 2.7 ± 1.3. There were 12 GRs, and 13 had PRs. Thus, neither V-HIT nor posturography predicted the prognosis. For c-VEMP, patients with GRs had significantly small AR concerning PR (19.2 ± 12.8 and 62.5 ± 42.5, respectively, [*p* < 0.01]). There were five normal, six unilateral, and 14 bilateral no response in 500hz o-VEMP. CGI-s of normal, unilateral, and bilateral no response was 1.4 ± 0.5, 2.8 ± 1.3, and 3.1 ± 1.2, respectively. There was a statistically significant difference between the normal and bilateral non-response o-VEMP groups (*p* < 0.05).

**Conclusion:**

Patients with VM had improvements in both headache and vertigo through a combination of lifestyle changes and prophylactic medications. Vestibular examinations, especially o- or c-VEMP, are beneficial for predicting the treatment outcomes of VM. The pathophysiology of VM is closely related to vestibular abnormalities, particularly the otolith-related pathways.

## 1 Introduction

Vestibular migraine (VM) is the most common cause of spontaneous episodic vestibular syndrome with a lifetime prevalence of approximately 1% (1). VM occurs in patients with a current or previous history of migraines and recurrent episodes of vestibular symptoms accompanied by migraine features. Treatment of VM is usually directed to the underlying condition by identifying and avoiding dietary triggers and prescribing prophylactic antimigraine medications (2). Antimigraine medications are considered when VM episodes are frequent, severe, or not well controlled by acute therapies; therapies should also be individualized based on comorbidities, potential side effects, and contraindications (2). Several studies have reported reduced or absent vestibular evoked myogenic potential (VEMP) responses in patients with VM indicating a dysfunction of the vestibulo-collic reflex (3–5). The pathophysiology of VM appears to be closely related to vestibular abnormalities, especially in the vestibulospinal pathways. Despite the increasing number of studies related to VM, most reports have focused on the characteristics of the disease, including the results of neurological tests (3–5). Treatment and prognosis related to vestibular abnormalities have not yet been fully documented. It is important to understand the prognostic factors to predict the disease course, properly counsel patients with VM, and conduct appropriate therapeutic planning. In our current study, we evaluated the response to medications in patients with VM with recurrent vertigo attacks for more than 1 month and determined the association between treatment responsiveness and abnormal vestibular results, including v-HIT (video-head impulse test), cervical and ocular vestibular evoked myogenic potentials (c- and o-VEMPs), and posturography measurements.

## 2 Methods

A retrospective chart review conducted from March 2020 to September 2022 identified 25 patients diagnosed with VM. The diagnosis of VM was based on the recent criteria for VM (4), which are as follows: (a) at least five episodes with vestibular symptoms of moderate or severe intensity, lasting 5 min to 72 h; (b) current or previous history of migraine with or without aura according to the International Classification of Headache Disorders criteria; and (c) one or more migraine features with at least 50% of vestibular episodes: 1. Headache with at least two of the following characteristics: one-sided location, pulsating quality, and moderate or severe pain intensity aggravated by routine physical activity; 2. Photophobia and phonophobia, 3. visual aura; and (d) not better accounted for by another vestibular or International Classification of Headache Disorders diagnosis. Other diseases that may cause recurrent vertigo, such as central origin or Meniere’s disease, and a previous history of otologic diseases or head trauma, were excluded from this study. There were 25 patients (24 females and one male). The average age was 44.2 ± 12.2 years.

Several questionnaires, including the Dizziness Handicap Inventory (DHI), Tinnitus Handicap Inventory (THI), and Hospital Anxiety and Depression Scale (HADS) were administered at the initial visit. Patients underwent pure-tone audiometry (PTA), c- and o-VEMP, v-HIT, and posturography during the interictal period, which was usually performed within one week of the first visit to the clinic.

### 2.1 Clinical symptom scale

#### 2.1.1 Dizziness handicap inventory

The DHI is a standard 25-question questionnaire designed to quantitatively evaluate the degree of handicap experienced by patients with vestibular disorders in their daily lives (6, 7). The total score ranges from 0 to 100, with 0 indicating no disability and 100 indicating severe disability.

#### 2.1.2 Tinnitus handicap inventory

The THI is a self-reported tinnitus handicap measure that is brief, easy to administer and interpret, broad in scope, and psychometrically robust (8). This is a self-reported measure that can be used in clinical practice to quantify the impact of tinnitus on daily living. The total score ranges from 0 to 100, with 0 indicating no disability and 100 indicating severe disability.

#### 2.1.3 Hospital anxiety and depression scale

The HADS is a questionnaire consisting of self-administered anxiety and depression subscales (9). Each HADS subscale is assessed using the seven questions (10). Each question was scored on a scale of 0 (not at all) to 3 (most of the time, very often). Therefore, the total score for each HADS subscale was 21 and the full HADS score was 42, with higher scores indicating higher levels of anxiety and depression.

### 2.2 Vestibular tests

#### 2.2.1 Video head impulse test

For vHIT testing, the right eye was recorded, and all three canals were evaluated (Otometrics ICS® Impulse) (11). During testing, the participants were fitted with goggles, seated, and asked to look at an eye-level target on a wall at a 1-meter distance. Following calibration, the examiner stood behind the patient, placed their hands on the participant’s head, and performed repeated head impulses randomized in velocity and direction in the plane of the tested semicircular canal. Head impulses (150–300°/s) were applied to each tested canal. Experienced practitioners performed all head impulses. The abnormality parameters were as follows: lateral canal VOR gain <0.8, vertical canal VOR gain <0.7, and/or the presence of corrective saccades (covert and/or overt) in any canal. The gain was calculated as the ratio of the area under the eye and head velocity curve (12).

#### 2.2.2 cVEMP

Electromyographic (EMG) signals were recorded using surface electrodes placed on the upper half of each sternocleidomastoid muscle (SCM) (active SCM), with a reference electrode placed on the lateral end of the upper sternum. In the supine position, the participants were asked to raise their heads to contract their SCM. The EMG signals were amplified and bandpass-filtered (20–2,000 Hz) using a Neuropack system (Nihon Kohden, Japan). Short-tone bursts (500 Hz air-conducted, 125 dB SPL; rise/fall time, 1 ms; plateau time, 2 ms) were used for stimulation at a repetition rate of 5 Hz. The analysis period was 100 ms (20 ms and 80 ms before and after the stimulus, respectively). Rectified EMG signals obtained during the prestimulation period were used to assess background muscle activity (13, 14).

The amplitude of p13-n23 (the first positive–negative deflection) was analyzed. The normalized amplitude (NA) was calculated as the p13-n23 amplitude divided by background muscle activity. Background muscle activity was calculated using rectified EMG signals obtained during the pre-stimulation period (−20 to 0 ms). The asymmetry ratios (AR) for the cVEMP were calculated as follows: AR = 100 × (NAl – NAs)/(NAl + NAs), where NAl represents the NA on the larger response side, and NAs represents the NA on the smaller response side. The upper limit of normal for AR was set at 41.6 (14). If no response was observed in either ear, AR was defined as 100. If AR was >41.6, the c-VEMP was defined as abnormal.

#### 2.2.3 oVEMP

The EMG signals were recorded using surface electrodes placed 1 cm below the center of each lower eyelid (active) and 2 cm below the active electrode (reference). During the recordings, the participants were instructed to maintain an upward gaze. Bone-conducted stimulation (500 Hz; rise/fall time, 1 ms; plateau time, 2 ms) was performed using a 4,810 mini-shaker (Bruel & Kjaer, Denmark) placed in the Fz position at a repetition rate of 5 Hz. The peak driving voltage was adjusted to 8.0 V, which produced a peak force level of 128 dB (re: 1 μN). The signals were amplified and bandpass filtered (20–2,000 Hz) using a Neuropack system. The raw amplitude of N1-P1 (the first negative–positive deflection) was analyzed. The upper normal limit for AR was set at 27.3 (AR) (13). If no response was observed in either ear, AR was defined as 100.

#### 2.2.4 Posturography

Gravicoda (ANIMA Corp., Tokyo, Japan) with eyes open and closed. The elliptical balance area(cm2) adopted in previous studies as a representative index of the degree of postural sway was used as an indicator for testing a solid surface. The data of environmental area (ENV; cm2) on the foam during the eyes-closed condition was used as an index of postural control (15). The absolute number of ENV cm2 was used for statistical comparison.

### 2.3 Treatment protocol and outcome assessments

Based on the literature (16, 17), lifestyle modifications and migraine prophylaxis have been used for treatment. Lifestyle modifications include regular exercise, sleep advice, avoidance of fasting, and potential dietary triggers. The same treatment were applied to the all subjects. Lomerizine 10 mg, amitriptyline 5 mg, and valproic acid 200 mg have been used as migraine prophylaxis agents. All of three were given to the patients in the initial visit.

Two weeks after the first visit, the patients were asked to evaluate the response and adverse events of the treatment. Two of the subjects did not want to keep taking amitriptyline and valproic acid due to the drowsiness and two of them stop taking them. All subjects could keep taking lomerizine. Four weeks after the first visit, the Clinical Global Impressions Scale (CGI-s) (18) was used to quantify the treatment response.

Each patient was further classified into two subgroups according to treatment outcomes with respect to vertigo and headache: CGI-S score from 0 to 2 (good response [GR]) and CGI-S score > 3(poor response [PR]).

This study protocol was approved by the Institutional Review Board of our institution.

#### 2.3.1 Statics

The results of v-HIT, c- and o-VEMPs, and posturography were compared between the two patient subgroups: GR and PR. Data were expressed as mean ± standard deviation. Continuous variables were compared using the Mann–Whitney U test. The Kruskal-Wallis test and Dunn’s Multiple Comparison test were used for multigroup comparisons. All statistical analyses were performed using GraphPad Prism, version 9 (GraphPad Software, San Diego, CA, United States). Statistical significance was set at *p* < 0.05 (Table 1).

**Table 1:** CGI-s.

CGI-S guidelines
1 = Normal—not at all ill, symptoms of disorder not present past seven days
2 = Borderline mentally ill—subtle or suspected pathology
3 = Mildly ill—clearly established symptoms with minimal, if any, distress or difficulty in social and occupational function
4 = Moderately ill—overt symptoms causing noticeable, but modest, functional impairment or distress; symptom level may warrant medication
5 = Markedly ill—intrusive symptoms that distinctly impair social/occupational function or cause intrusive levels of distress
6 = Severely ill—disruptive pathology, behavior and function are frequently influenced by symptoms, may require assistance from others
7 = Among the most extremely ill patients—pathology drastically interferes in many life functions; may be hospitalized

## 3 Results

**Figure 1:**
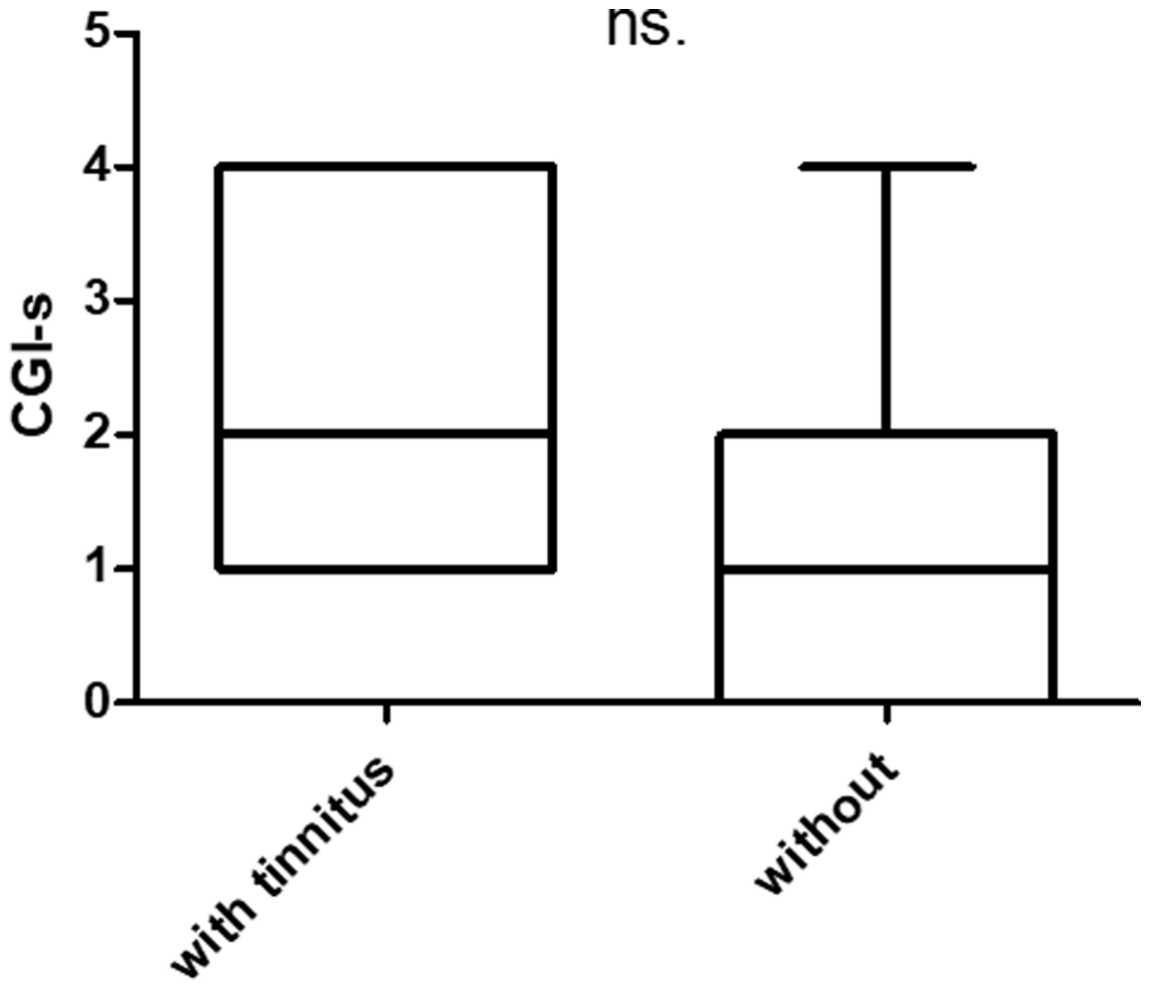
Prognosis of patients with and without tinnitus. THI (tinnitus handicap inventory). There are 17 patients with tinnitus (THI = 0) and eight without tinnitus (THI = 21.0 ± 18).

Overall, after treatment, most of the patients had improved dizziness and headache, and the CGI-s was 2.7 ± 1.3. Twelve and 13 participants had GR and PR, respectively. Participant characteristics based on treatment responses are shown in Table 2. There were no statistically significant differences between the two groups (n.s). Tinnitus was present and absent in 17 and 8 patients, respectively. The treatment differences between patients with and without tinnitus are summarized in Figure 1. The score of THI was 21.0 ± 18.1 in patients with tinnitus. There were no statistically significant intergroup differences in treatment outcomes between the two groups.

**Table 2:** Subjects characteristics based on treatment response.

	Age (average ± SD)	Male	Female	CGI-S	DHI total	HADS total
GR(CGI-S less than 2)	43.8 ± 13.0	0	12	1.5 ± 0.5	46.8 ± 17.6	15.8 ± 9.9
PR(CGI-3 more than 3)	44.6 ± 12.0	1	12	3.7 ± 0.9	44.6 ± 12.1	16.5 ± 7.3
Total	44.2 ± 12.3	1	24	2.72 ± 1.3	45.7 ± 14.7	16.2 ± 8.5

There are no statistically significant difference.

**Figure 2:**
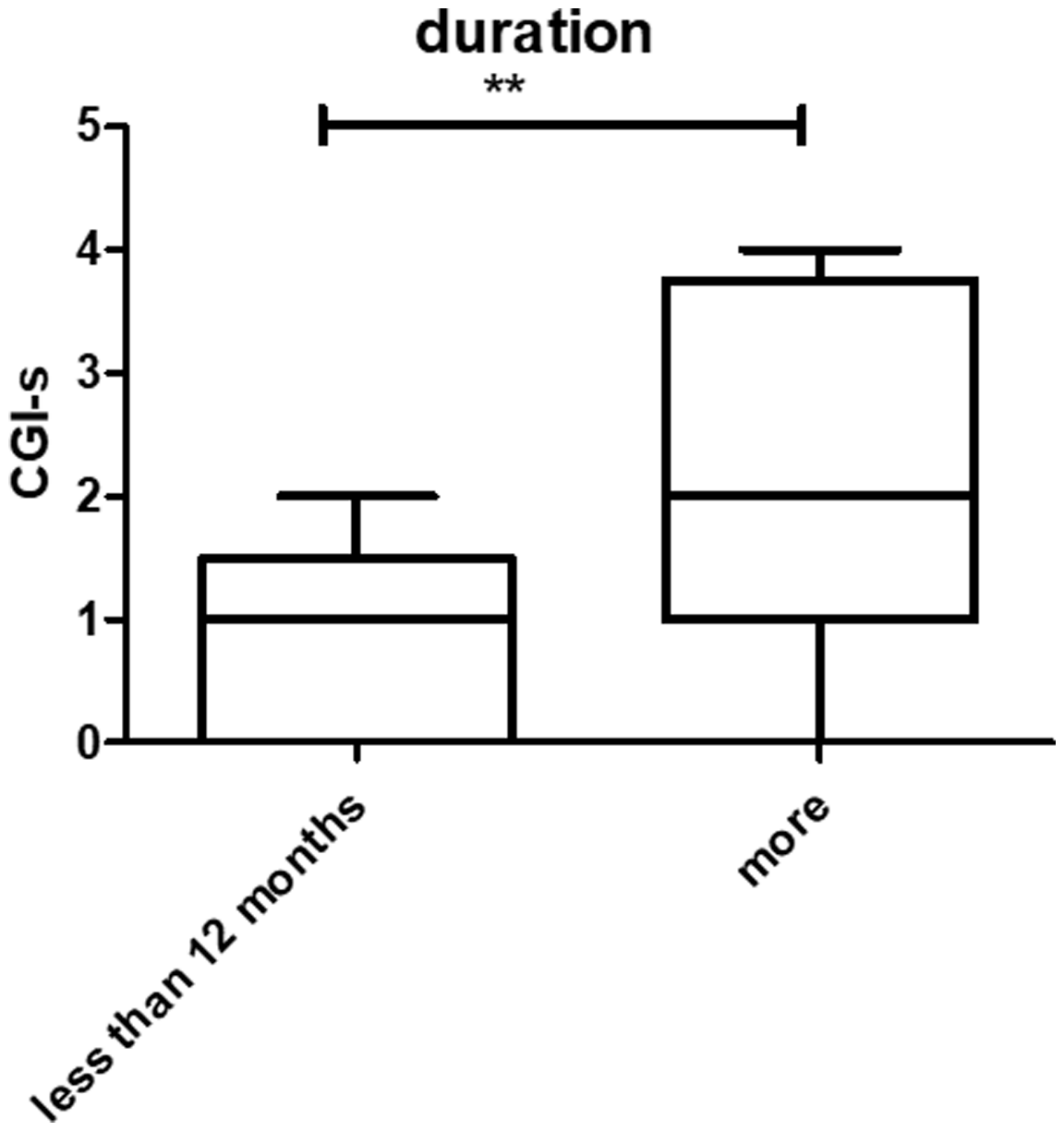
Prognosis and duration of the disease. There were statistically significant differences between patients who had symptoms at <12 months and > 12 months (**p < 0.01).

The disease duration ranged from 1 month to 480 months. The average was 75.2 ± 105.7 months. The CGI-s was 1.77 ± 0.8 and 3.2 ± 1.3 in patients who suffered less than 12 months and more than 12 months, respectively. A statistically significant difference was observed between the two groups (*p* < 0.001) (Figure 2).

Regarding the semicircular canal function, there were eight normal and 17 abnormal VHITs. There were no statistically significant differences between the GR and PR groups as shown in Table 3.

**Table 3:** VHIT and prognosis.

	Normal VHIT	Abnormal VHIT	Total
GR (CGI-S less than 2)	4	8	12
PR (CGI-3 more than 3)	4	9	13
	8	17	25

There are no statistically significant difference.

**Figure 3:**
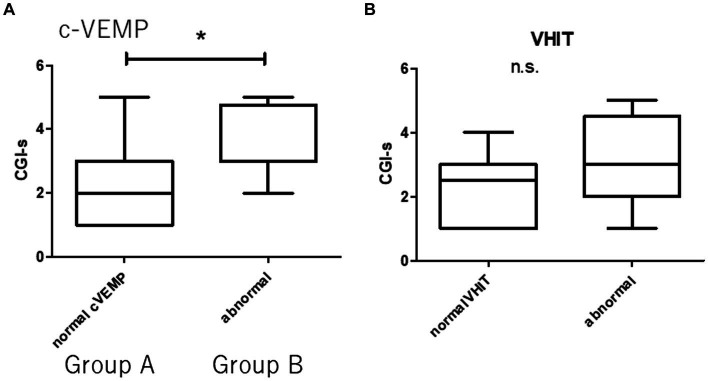
Prognosis and vestibular dysfunction. Group A (normal c-VEMP). Group B (abnormal c-VEMP). There are 17 patients in Group A and eight in Group B. There are 9 abnormal VHIT and 8 normal VHIT. There are 8 abnormal VHITs in Group B. **(A)** Statistically significant difference between patients with normal and abnormal c-VEMP (*p < 0.05). **(B)** There are no statistically significant differences between patients with normal and abnormal VHIT (n.s., not significant).

Concerning c-VEMP, patients with GR were significantly small AR with respect to PR [19.2 ± 12.8 and 62.5 ± 42.5, respectively, (*p* < 0.01)]. There were 17 cases of normal c-VEMP and 8 cases of abnormal c-VEMP. Group A was defined as normal c-VEMP, and Group B was defined as abnormal c-VEMP. The patients of Group A had better prognosis (CGI-s = 2.3 ± 1.3 and 3.5 ± 1.1 respectively) (Figure 3), with 9 abnormal VHIT and 8 normal VHIT participants. There were eight abnormal VHIT cases in group B. Statistically significant differences were observed between the two groups concerning the VHIT results (*p* < 0.05). Participants had 8 normal VHIT, and 17 had an abnormal VHIT. Concerning treatment outcome, CGI-s of normal VHIT and abnormal VHIT were 2.2 ± 1.2 and 2.9 ± 1.4, respectively. There were no statistically significant differences between the two groups (Figure 3).

**Figure 4:**
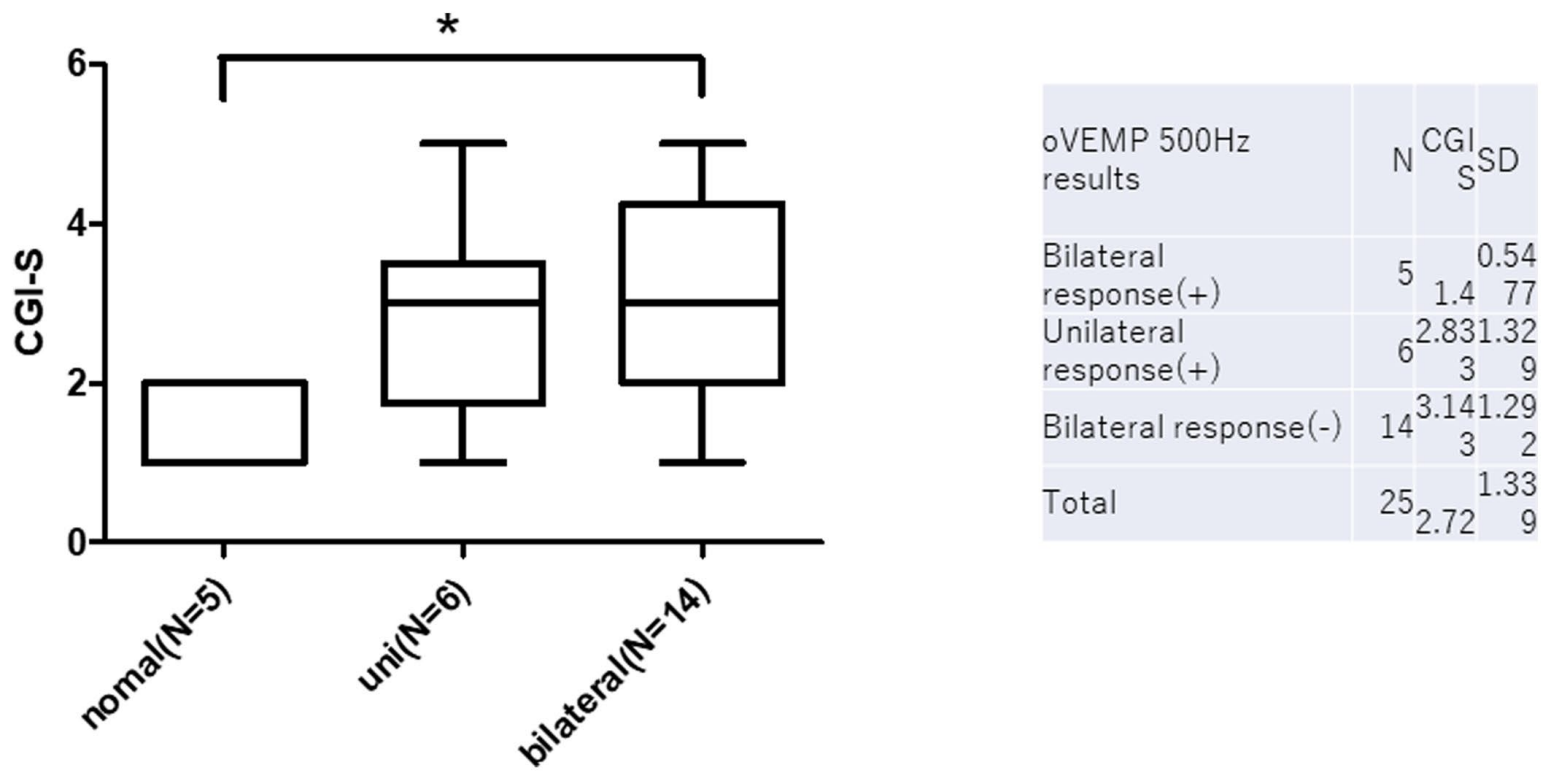
O-VEMP 500 Hz results and prognosis. There are statistically significant differences between normal and bilateral no responses in c-VEMP on CGI-s (*p < 0.05).

oVEMP 500 Hz results and prognosis are summarized in Figure 4. There were five normal, six unilateral, and 14 bilateral no responses in 500hz o-VEMP. CGI-s of normal, unilateral, and bilateral no responses were 1.4 ± 0.5, 2.8 ± 1.3, and 3.1 ± 1.2, respectively. There was a statistically significant difference between normal and bilateral no responses (*p* < 0.05).

**Figure 5:**
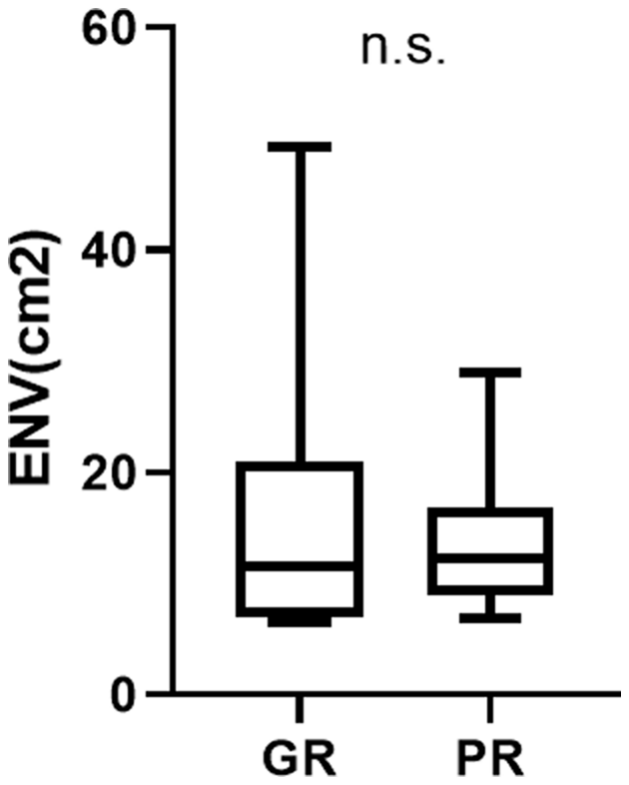
The result of posturography and prognosis (environmental area) ENV area cm2 represents the environmental area (cm2) on the foam rubber under eyes-closed conditions. There is no statistically significant difference between the GR and PR groups in the posturography results (n.s.).

The Prognosis and posturography results are summarized in Figure 5. The ENV cm2 on the foam rubber during the eyes-closed condition was 15.7 ± 12.3 in GR and 13.5 ± 6.0 in PR. There was no statistically significant difference between the GR and GR (n.s.) (Figure 5).

## 4 Discussion

In our study, most patients experienced improvements in headache and dizziness, as evaluated by the CGI-s at 4 weeks after treatment. These results are similar to those of a previous study (19), in which more than 70% of patients with VM experienced improvements in both headache and vertigo through a combination of lifestyle changes and prophylactic medications. They concluded that abnormal vestibular ratios on posturography and abnormal VEMP responses were frequent findings in patients with VM with recurrent attacks for more than 6 months and were indicators of a poor prognosis.

Vestibular examinations revealed 17 abnormal VHIT, eight abnormal c-VEMPs, and 20 abnormal o-VEMPs. Among them, o- and c-VEMP results were related to treatment outcomes.

We attempted to identify the predictive factors in terms of prognosis in our VM cases by dividing them into two subgroups according to treatment response and comparing the results of vestibular tests between these two groups. Although the VHIT results were not significantly different between groups, abnormal o- or c-VEMPs were indicators of poor treatment outcomes. Furthermore, patients with normal c-VEMP levels have significantly better treatment outcomes. The results of o and c-VEMP were significantly better in patients with GR than in those with PR. But there was no significant difference of the ENV between patients with GR and with PR. Usually, patients with bad result of o and c-VEMP show bad result of ENV. If we see the relation between the result of c- and o-VEMP, and posturography, bad result of o and c-VEMP show bad result of ENV as we expected. However not all the subjects in GR bad good results of c- and o-VEMP.

Two of the subjects in GR group had significantly bad resus of posturography as we can see the large standard deviation in the Figure 5. These two subjects were exceptionally good prognosis with but bad c-and o-VEMP results. This error is related the small number of the subjects.

These findings suggest that the prognoses of headache and vertigo in VM are closely related to abnormalities in the vestibulospinal pathways, which may play a role in the pathogenesis of this disorder.

In previous reports, vestibular tests have focused on the detection of abnormal vestibuloocular function in the lateral semicircular canal. The C-VEMP has been used as a clinical test for vestibulospinal function, particularly in the saccule and inferior vestibular nerve regions. It has been reported that the rate of absence of unilateral or bilateral c-VEMP response was present in 44% of patients with VM compared to 3% of healthy controls and that c-VEMP amplitudes were significantly reduced in VM compared to controls, suggesting that peripheral vestibular structures, such as the saccule and central vestibular structures, are affected, and the inner ear also seems to contribute to vertigo in VM (3, 20).

In the present study, 32% (8/25) of patients who underwent c-VEMP tests showed abnormal results, and patients with normal c-VEMP showed significantly better recovery. These results are similar to those of a previous study, in which 29% of patients with abnormal c-VEMP had poor recovery compared to those with normal c-VEMP (19).

An important question that arises from the current study and findings of previous reports is the role of vestibular abnormalities in VM. There are many hypotheses concerning how migraines may result in audiovestibular symptoms. Audiovestibular symptoms can arise from trigeminal neurogenic inflammation of the labyrinth, resulting in local plasma extravasation or vasospasm of the internal auditory artery (21). The reciprocal connections between the inferior, medial, and lateral vestibular nuclei and the trigeminal nucleus caudalis suggest that vestibular and trigeminal information processing may be altered concurrently during migraine attacks and that vestibular signals may directly influence the trigeminovascular reflex pathways (22). However, it was reported that comorbid conditions (Meniere’s disease, benign paroxysmal positional vertigo, chronic subjective dizziness) are important contributors to vestibular symptoms and that induced vertigo can act as a migraine trigger in patients with migraine (23–25).

The present study had several limitations. First, the number of the subjects were too small to reach the conclusion. We should confirm the results with further study. Second, we used CGI-S for the scale. The best scale in this study would be a comparison of pre and post DHI score. However, since our study is retrospective chart review based on the medical record on daily practice, post DHI was not obtained. We usually recorded the CGI-S in daily practice, we used this scale for clinical evaluation. Third, we defined four weeks for the evaluation of treatment results, which may be brief. Evaluating the optimal medications for VM requires a double-blind, placebo-controlled, randomized study because of the complex nature of this condition. Forth, we cannot tell whether interictal test abnormalities reflect underlying deficits in the periphery or central mechanisms or vulnerabilities that can trigger subsequent attacks. Forth, an abnormality in the o-and c-VEMP tests does not indicate an abnormality of the vestibule, and the motor and central nervous systems can also influence the results. Despite these drawbacks, we evaluated the effectiveness of the current treatment strategy for VM and identified factors associated with the prognosis of this disorder using the same treatment strategy. Further studies with larger populations are needed to establish this exact relationship.

## 5 Conclusion

1. The 25 patients with VM were conventionally treated. Four weeks after treatment, CGI-s were used to quantify the treatment response. There were 12 GRs, and 13 had PRs.
2. There are no statistically significant differences in the score of questionnaires including DHI and HADS between the GR and PR groups.
3. The worse prognosis was observed in patients suffering from VM for more than 12 months (*p* < 0.001).
4. The results of V-HIT, and posturography did not influence the treatment outcome.
5. Better prognosis was observed in patients with the presence of bilateral o-VEMP or AR <40 of c-VEMP.
6. Both o- and c-VEMP may be useful in predicting the prognosis in patients with VM.

## Data availability statement

The raw data supporting the conclusions of this article will be made available by the authors, without undue reservation.

## Ethics statement

The studies involving humans were approved by Research Ethics Committee, Tokai University School of Medicine. The studies were conducted in accordance with the local legislation and institutional requirements. The participants provided their written informed consent to participate in this study.

## Author contributions

FG: Writing – original draft, Writing – review & editing. KW: Writing – review & editing. SK: Writing – review & editing. KO: Writing – review & editing.
